# Wearable Power-Assist Locomotor for Gait Reconstruction in Patients With Spinal Cord Injury: A Retrospective Study

**DOI:** 10.3389/fnbot.2022.775724

**Published:** 2022-02-18

**Authors:** Soichiro Koyama, Shigeo Tanabe, Takeshi Gotoh, Yuta Taguchi, Masaki Katoh, Eiichi Saitoh, Yohei Otaka, Satoshi Hirano

**Affiliations:** ^1^Faculty of Rehabilitation, School of Health Sciences, Fujita Health University, Toyoake, Japan; ^2^Department of Rehabilitation, Fujita Health University Hospital, Toyoake, Japan; ^3^Department of Rehabilitation Medicine I, School of Medicine, Fujita Health University, Toyoake, Japan

**Keywords:** wearable robotic exoskeleton, gait, paraplegia, tetraplegia, clinical experience

## Abstract

Wearable robotic exoskeletons (WREs) have been developed from orthoses as assistive devices for gait reconstruction in patients with spinal cord injury. They can solve some problems encountered with orthoses, such as difficulty in independent walking and standing up and high energy consumption during walking. The Wearable Power-Assist Locomotor (WPAL), a WRE, was developed based on a knee–ankle–foot orthosis with a single medial hip joint. The WPAL has been updated seven times during the period from the beginning of its development, in 2005, to 2020. The latest version, launched as a commercialized model in 2016, is available for medical facilities. In this retrospective study, which included updated results from previous reports, all data were extracted from development research records from July 2007 to December 2020. The records were as follows: patient characteristics [the number of participants, injury level, and the American Spinal Injury Association Impairment Scale (AIS) score], the total number of WPAL trials when aggregating the cases with all the versions or only the latest version of the WPAL, and maximum walking performance (functional ambulation category [FAC], distance, and time of continuous walking). Thirty-one patients participated in the development research. The levels of spinal cord injury were cervical (C5–C8), upper thoracic (T3–T6), lower thoracic (T7–T12), and lumbar (L1) in 10, 5, 15, and 1 of the patients, respectively. The numbers of patients with AIS scores of A, B, C, and D were 20, 7, 4, and 0, respectively. The total number of WPAL trials was 1,785, of which 1,009 were used the latest version of the WPAL. Twenty of the patients achieved an FAC score of 4 after an average of 9 (median 8, range 2–22) WPAL trials. The continuous walking distance and time improved with the WPAL were compared to the orthosis. We confirmed that the WPAL improves walking independence in people with a wide range of spinal cord injuries, such as cervical spinal cord injuries. Further refinement of the WPAL will enable its long-term use at home.

## Introduction

Traumatic spinal cord injury (SCI) is one of the most devastating events that occur after various accidents. Long-term motor, sensory, and autonomic dysfunction caused by traumatic SCI have a tremendous effect on the daily life of patients and their families. The incidence rates of traumatic SCI per 1 million people per year were 14 cases in Austria (Majdan et al., 2016), 18 cases in Switzerland (Chamberlain et al., 2015), and 10 cases in Denmark (Bjornshave Noe et al., 2015), representing almost 180,000 cases annually worldwide in 2014 (Lee et al., 2014). Similarly, the annual incidence of SCI is approximately 54 cases per 1 million people in the United States, which is approximately 17,900 new SCI cases in 2021 (NSCIS Center, 2021). In Japan, the estimated incidence of traumatic SCI, excluding the grade E in the Frankel scale, that is, defined as no neurological deficit/complete recovery, was 49 cases per 1 million people annually in 2010 (Miyakoshi et al., 2020).

The majority of patients with motor-complete SCI often rely on a wheelchair as a mobility device in activities of daily living because a wheelchair is energy efficient and enables patients to safely perform their daily activities. However, long-term inactivity due to wheelchair use results in various medical problems [e.g., joint contraction (Kunkel et al., 1993), pressure sores (Verschueren et al., 2011), osteoporosis (Varacallo et al., 2021), and psychosocial problems as a result of a relatively low eye level (Dijkers, 1999; Levins et al., 2004)]. For patients with SCI, the opportunity to stand and walk occasionally is important from both physical and psychosocial perspectives.

In the past few decades, various wearable robotic exoskeletons (WREs) have been developed for stand and gait reconstruction in patients with motor-complete SCI. They offer the opportunity to walk in home and community environments by moving the paretic legs of patients with partial or complete SCI in a reciprocal stepping pattern (Fisahn et al., 2016; Miller et al., 2016; Palermo et al., 2017; Tan et al., 2021). Arazpour et al. (2013) reported that the gait, speed, and endurance of patients with SCI using WREs are superior to those of patients using either reciprocating gait or hip–knee–ankle–foot orthoses (Arazpour et al., 2013). The oxygen consumption and heart rate during gait training with WRE are slightly increased compared with that during sitting and standing, and the load during gait training with WRE is less than that with conventional orthoses (Asselin et al., 2015; Yatsuya et al., 2018).

We have previously reported the effects of the Wearable Power-Assist Locomotor (WPAL) on walking ability and gait pattern in patients with various levels of SCI. The first report shared the basic concept of WPAL development and a comparison of walking performance between the WPAL and the conventional Primewalk orthosis (Tanabe et al., 2013b). The report showed that the WPAL has a lower physiological cost index and involves less muscle activity in the upper limbs during walking, compared with the Primewalk orthosis (Tanabe et al., 2013b). We also reported gait pattern, the basic training procedure, and gait performance of the WPAL in seven patients with SCI at the thoracic level of injury (T6–T12) (Tanabe et al., 2013a). In addition, we found that continuous walking time and distance were prolonged with the WPAL compared with orthotic walking in 12 patients with SCI (Hirano et al., 2015). The WPAL significantly decreased the physiological cost index, heart rate, and modified Borg score during the 6-min walking test compared with conventional knee–ankle–foot orthoses in six other patients with SCI (Yatsuya et al., 2018). Recently, we have reported that the WPAL improves walking ability more than conventional orthoses in patients with cervical SCI (Fuse et al., 2019).

The objective of this study was to summarize all data that include updated results from previous reports, about physical characteristics, and walking abilities of patients with SCI, from the development research records in clinical practice from July 2007 to December 2020. The present findings are potentially useful for gait performance comparison with other robots, a meta-analysis of the effects of WREs in patients with SCI, and evidence-based selection of WREs for patients with SCI.

## Materials and Methods

### Study Design and Participants

In this retrospective study, we included 31 patients with SCI in our university from 2007 to 2020, regardless of being inpatients or outpatients. The causes of spinal cord injury were traumatic spinal cord injury in 24 patients, spinal cord infarction in 3 patients, encephalomyelitis in 1 patient, radiation myelitis in 1 patient, hemorrhage from the thoracic spinal cord cavernous hemangioma in 1 patient, and acute thoracic spinal epidural hematomas in 1 patient. The principal inclusion criteria for participation were as follows: (1) patients with motor paralysis [American Spinal Injury Association Impairment Scale (AIS) classification A–C] who had a neurological level of injury from C3 to L1; (2) a height of 155–180 cm; (3) a weight <80 kg; and (4) sufficient upper limb muscle strength (to the extent that the patient can transfer independently). All patients provided written informed consent prior to study participation. This study was performed in accordance with the principles of the Declaration of Helsinki and conducted after receiving approval from the ethics committee of our university (approval number: CR18-035).

The exclusion criteria were as follows: (1) progressive disease (excluding disuse syndrome); (2) difficulty in communication due to dementia or impaired consciousness; (3) high risk of fracture of the lower limbs or spine (e.g., severe osteoporosis); (4) uncontrolled hypertension (resting systolic blood pressure >180 mmHg and diastolic blood pressure >120 mmHg); (5) uncontrolled tachycardia (ventricular rate >120 beats per min); and (6) limitation of movement due to impaired cardiac or respiratory function (e.g., shortness of breath when using a wheelchair).

### Design of WPAL

A detailed basic design of the WPAL has been published previously (Tanabe et al., 2013a,b, 2017). The main structures, such as frames and motors, were placed between the lower limbs. The frame was connected by a single mechanical hip joint medially under the perineum. The mechanical hip joint had a sliding structure that curves anterior-posteriorly based on the structure of Primewalk orthosis (Suzuki et al., 2005). The sliding structure enables the virtual center of rotation of the robotic hip joint to be closer to the physiological center of the hip joint. Six motors were located in the hip, knee, and ankle joints. The ranges of motion were as follows: hip, 40° (flexion 25°-extension 15°); knee, 120° (flexion 120°-extension 0°); and ankle, 50° (dorsiflexion 35°-plantar flexion 15°). Each joint used an individual custom-made brushless DC servomotor, which was compact to fit between the legs (24 V, 78 W, peak torque of 4 Nm, speed range from 0 to 1,000 deg/s). The weight of the WPAL was approximately 13 kg; however, the patient did not feel the weight because one foot was always on the ground. The use of a customized walker with motor control circuitry and batteries ensured safety and eliminated the need for the patients to carry the device themselves. Two lever switches and two button switches were installed on both handgrips of the walker to enable the patients to operate it themselves. The WPAL could also be put on and removed by the patient; a skilled patient could do this within approximately 2 min.

### Operating the WPAL

**Figure 1:**
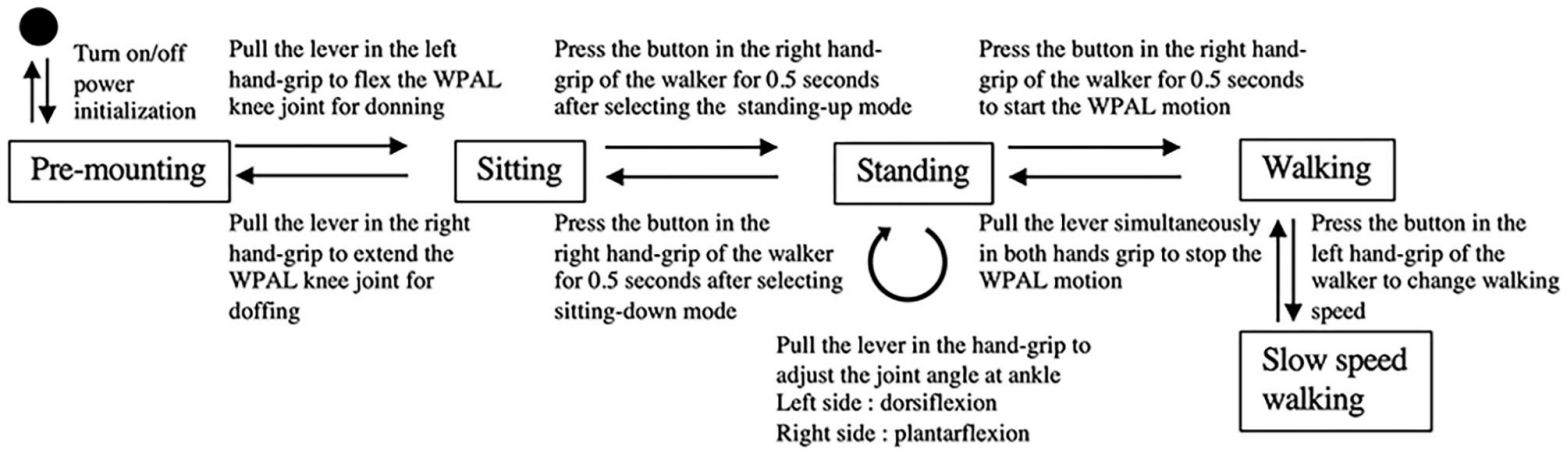
State transition diagram for the WPAL operation. WPAL, Wearable Power-Assist Locomotor.

The WPAL is equipped with the following five modes: (1) standing-up mode, (2) adjustment mode for the ankle joint angle during standing, (3) walking mode, (4) sitting-down mode, and (5) manipulation mode for the knee joint while putting on or removing the WPAL. Each mode is selected by pressing the button attached to the left grip of the walker. When the WPAL user presses a button, only the indicator lamp for the currently selected mode lights up on the control panel mounted on the front bar of the walker to indicate the currently selected mode. Figure 1 shows a state transition diagram for the WPAL operation and control.

In the standing-up mode, pressing the button attached to the right grip of the walker for 0.5 s will start the WPAL in motion from the sitting position. The user stands up by moving the body's center of gravity slightly forward and pushes the walker slightly downward and backward using residual functions in accordance with the movement of the WPAL. In the adjustment mode for the ankle joint angle during standing, the user can adjust the ankle joint angle for a stable standing posture by pulling the lever attached below the grip of the walker. Pulling the lever on the left side increases the dorsiflexion angle of the ankle joint, and pulling the lever on the right side increases the plantar flexion angle of the ankle joint. This function is necessary because each patient with SCI has a different ankle joint angle for a stable standing posture. In the walking mode, the WPAL motion is started by pressing the button attached to the right grip of the walker for 0.5 s. In this mode, the user is able to select the first step of walking (left or right). If the user selects the left side for the first step, the user has to shift the center of gravity to the right side to start walking smoothly. The WPAL moves both lower limbs alternately and constantly. WPAL users have to move their center of gravity laterally rhythmically with the WPAL motion using residual upper limb and trunk muscles and move the walker forward at the appropriate time in the gait cycle of the WPAL. WPAL motion is stopped when the user pulls the levers on both sides simultaneously for a few seconds. In the sitting-down mode, the WPAL motion is started by pressing the right button for 0.5 s in the standing position. The sitting-down motion of the WPAL is caused first by plantar flexion of the ankle joint, followed by flexion of the knee joint. The user needs to lean forward with their trunk and maintain the posture until full flexion to the pre-programmed angle of the knee joint of the WPAL for sitting in the wheelchair. In the manipulation mode of the knee joint for putting on and removing the WPAL, the user can manipulate the knee joint of the WPAL by using the lever attached under the grip of the walker. The user flexes the knee joint of the WPAL by pulling the lever under the left grip. This action is used when the user wants to put on the WPAL from a wheelchair. In contrast, pulling the right lever extends the knee joint of the WPAL. This action enables the user to remove the WPAL while on the wheelchair after the sitting-down motion.

### Gait Training Procedure

The WPAL moves both lower limbs in a constant rhythm. WPAL users need to shift their lateral weight rhythmically in accordance with the WPAL motions. We have recommended five stages of gait training to achieve independent walking with the WPAL and a specialized wheeled walker (Tanabe et al., 2013a,b, 2017). In the initial four stages of gait training, the exercises are performed under the suspension system to prevent falls and reduce excessive fear of falling. The harness of the suspension system is set to slack without partially supporting the body weight. The first stage is a stepping exercise in the parallel bars. Before walking with the WPAL, the user learns to use the upper limbs and trunk muscles to perform a lateral weight shift with appropriate timing while keeping their center of gravity forward and backward, referring to a beeping sound produced in time with the walking rhythm. Subsequently, the user performs the same motions as the driving WPAL. The second stage is walking in the parallel bars. The step time is gradually shortened to approximately 1.5 s. The stride length is gradually increased to approximately 200 mm. The third stage is a walking exercise on a treadmill with a slow speed (approximately 0.1–0.3 km/h). Through continuous walking for a long period, the WPAL user can learn stable and rhythmic lateral weight shifts. The fourth stage is a walking exercise with a specialized wheeled walker. The user operates the WPAL using buttons or triggers installed on the grip of the walker. The final stage is a walking exercise without suspension using a specialized wheeled walker. Consensus decision-making is made between a certified physical therapist and a rehabilitation physician as to whether to proceed to the next stage. Patients also practice standing up and sitting down and donning/doffing of the WPAL during trials.

### Data Collection and Analysis

The development history of the WPAL was obtained from research records and interviews with members of the WPAL development team (rehabilitation physician, physical therapist, prosthetist and orthotist, and engineer). In the research records, medical doctors or physical therapists in the team recorded, on a daily basis, the details of training, any troubles related to the training as well as the robots, and all other necessary information, such as the version of the robots. Interviews were conducted to confirm the contents of the research records if needed. The number of WPAL trials was calculated from the detailed trial data from 2007 to 2020 in the development research records. However, the WPAL trials included a few exceptional cases where the trial did not contain gait training with the WPAL; instead, the trial was used for fine-tuning the WPAL motion and three-dimensional gait analysis with the WPAL and/or the orthosis only. If the name of the patient was not recorded, we included only the total number of WPAL trials. The number of WPAL trials for each patient was calculated for those whose names were listed in the detailed trial data. The number of WPAL trials to reach a functional ambulation category (FAC) score of 4 was also counted from the development research records. The reasons for the discontinuation of the WPAL trials were also collected from the development research records. Motor function was assessed using the AIS score. The evaluation was performed by a rehabilitation physician and the physical therapist in charge, and the score was agreed upon by both parties. Gait performance using the WPAL was measured in terms of the maximum continuous walking distance and time. However, the maximum run time of the WPAL is 120 min owing to the battery capacity. The Kolmogorov-Smirnov test was used to evaluate the pattern of data distribution. Since the normality of the data was not confirmed, the FAC score and maximal continuous walking distance and time were compared between the orthosis and WPAL using the Wilcoxon signed-rank test. The FAC score was used for all patients with both orthotic and WPAL records, and the maximum continuous walking distance and time were used for patients who reached an FAC score of 4 only. Adverse events, such as falls, were recorded by a certified physiotherapist who supervised the patient while walking with the WPAL. The patients were divided into four groups according to the level of injury: cervical (C5–C8), upper thoracic (T1–T6), lower thoracic (T7–T12), and lumbar. If the SCI level of the patient was different between the left and right sides, the higher level was defined as the injury level. The practice was performed for 1 or 1.5 h per day, i.e., preparatory exercises. All statistical analyses were performed with SPSS version 25 (SPSS Inc., Chicago, IL, USA). Any values of *p* < 0.05 were considered statistically significant.

## Results

### Development History of WPAL

**Figure 2:**
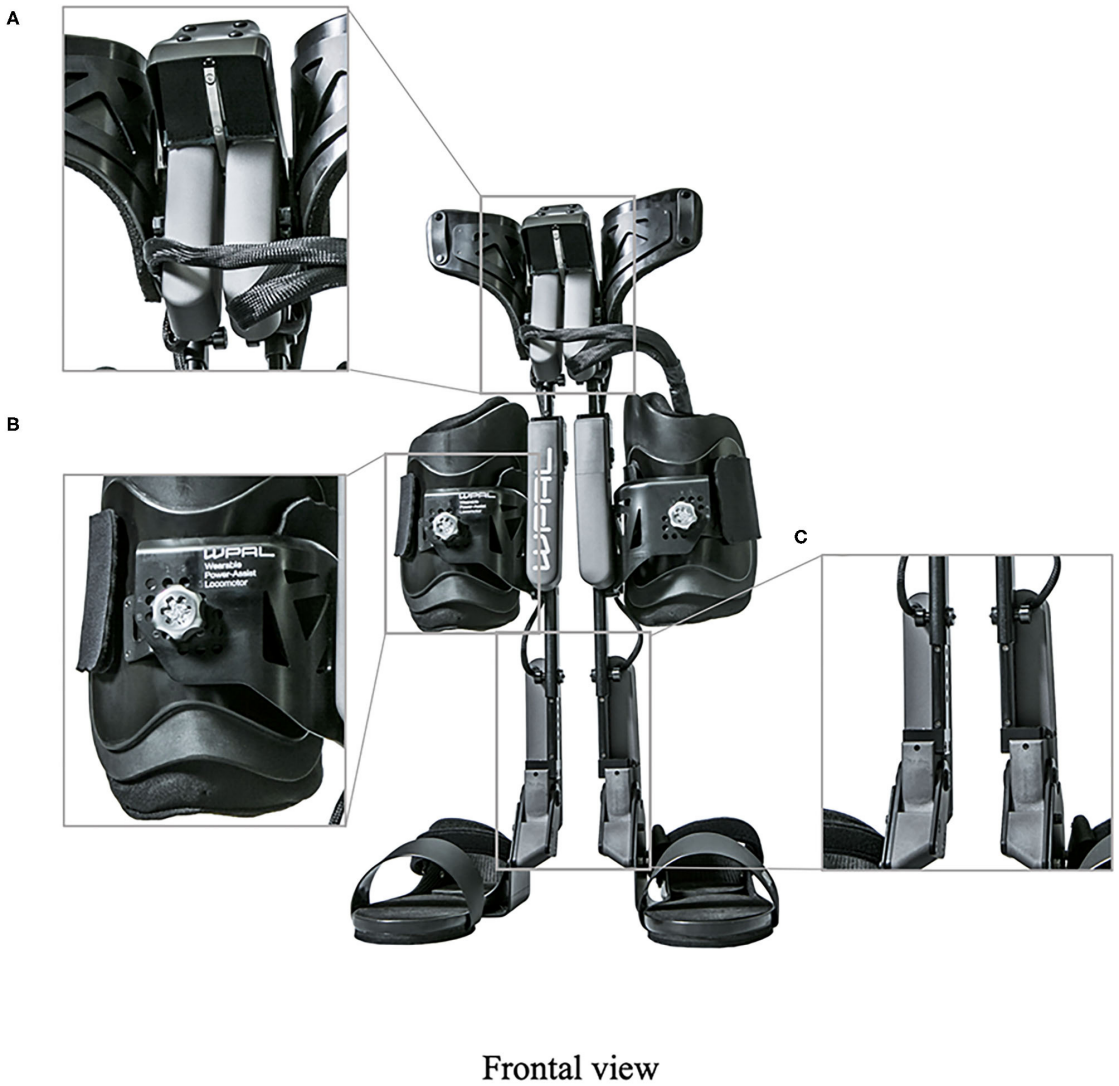
The structure of the latest WPAL in detail. **(A)** The hip joint in frontal view, **(B)** the knee joint in frontal view, and **(C)** the ankle joint in frontal view. WPAL, Wearable Power-Assist Locomotor.

The WPAL, developed in collaboration with Aska Corporation and Tomei Brace Co. in 2005, is a walking-assist robot for people with SCI, based on the design concept of a knee–ankle–foot orthosis with a single medial hip joint (Primewalk) (Suzuki et al., 2005) (Table 1 and Figure 2). From the Primewalk, which was the basis of the WPAL, to the commercialized current model, seven major updates were implemented to improve the design and control system. The first version was a Primewalk with a total of five motors attached one to the hip joint, two to both knee joints, and two to both ankle joints. Six months later, a hip joint motor was mounted on both sides for a total of six motors in the second version. In 2007, the motors were newly developed with the support of the New Energy and Industrial Technology Development Organization in the third version. In 2008, the thigh and lower leg cuffs were made smaller and lighter. The orthotic parts were improved so that the thigh and lower leg cuffs were independent in the fourth version. When patients with SCI used the WPAL to walk, the orthotic parts were first attached to the thigh and lower leg cuffs; then, they were connected to the robotic part by themselves. This connecting procedure between the cuff and robotic part was the same as that in the current system. The shape of the footplate was also improved. In 2010, in the fifth version, the motor was improved by attaching a cover. In 2011, we developed a universal cuff and improved the positioning of the robot and the cuff fixation part in the sixth version. In 2016, the seventh and current WPAL model began commercialization for medical and social service organizations. The touchscreen tablet PC for control and settings was placed in front of the walker. Until August 2007, we used the direct current (DC) brush motors manufactured by Maxon of Switzerland. Then, we developed a new brushless servo motor that had two advantages for our robot. First, the motor allows long-term use because of better heat dissipation and lower friction. Second, it can be installed between both the lower limbs. Consequently, we introduced minor updates (mainly improvement in heat resistance) in the motor. The currently used dedicated motor can never be damaged by heat.

**Table 1:** Development history of the Wearable Power-Assist Locomotor (WPAL).

**Version**	**0**	**1**	**2**	**3**
Date	4/1998	11/2005	3/2006	8/2007
Feature	Primewalk	• Base on primewalk • Five commercially motors (1 hip, 2 knee, 2 ankle)	• Base on primewalk • Six commercially motors (2 hip, 2 knee, 2 ankle)	• Development of specialized motors • Improvement of orthotics parts
Picture	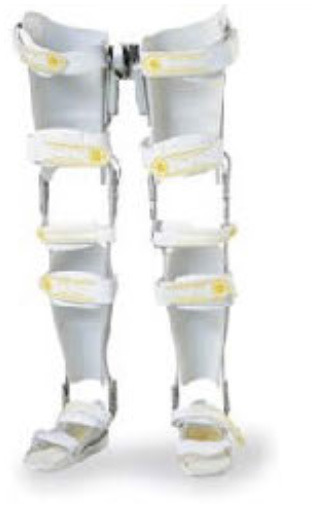	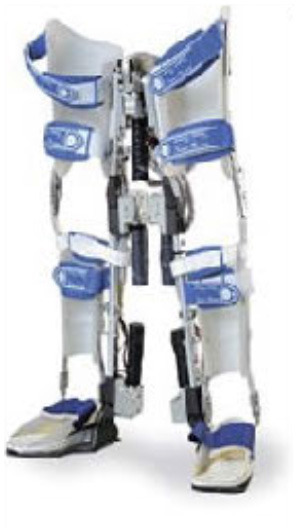	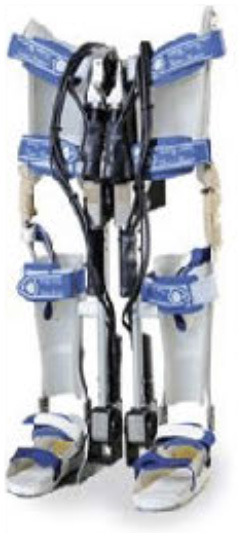	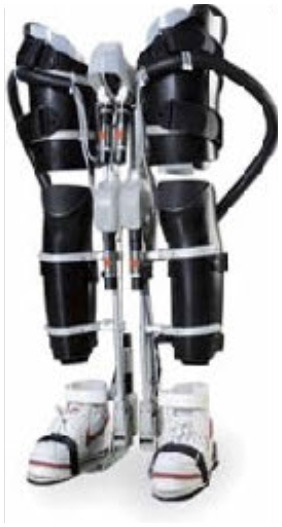
**Version**	**4**	**5**	**6**	**7**
Time	1/2008	7/2010	12/2011	10/2016
Feature	• Miniaturization of femoral and lower leg cuff • Change in the don/doff method • Improvement of foot shape	• Development of motor cover and control box • Improvement of servo motor • New servo amplifier	• Development of universal cuff • cuff mounting bracket adjustment mechanism • Adjustable leg length	• New design • Improved casting module • Improved motor • New display unit • Adoption of tablet
Picture	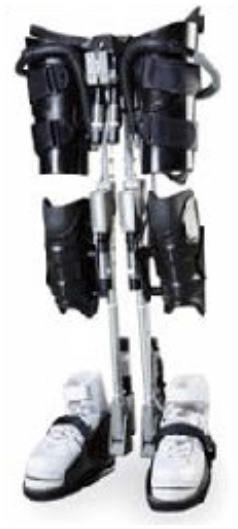	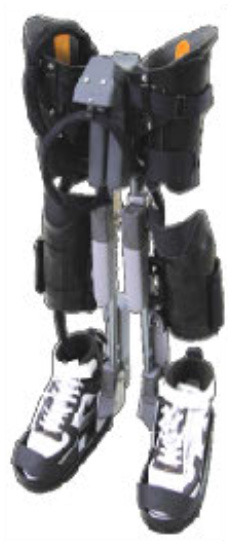	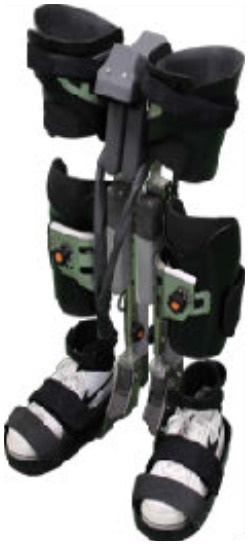	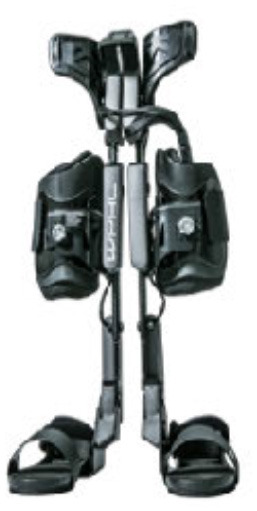

### Number of WPAL Trials

The SCI level was cervical (C5–C8) in 10 patients, upper thoracic (T3–T6) in 5 patients, lower thoracic (T7–T12) in 15 patients, and lumbar (L1) in one patient. The proportions of patients with AIS scores of A, B, C, and D were 20, 7, 4, and 0, respectively (Table 2). The total number of WPAL trials was 1,785, of which 1,009 used the latest WPAL version, and the names of the patients were not recorded in the 88 development research records. Three falls due to mechanical errors in the battery control system and the servo motor system associated with lower limb spasticity in patients with SCI were observed. No severe incidents, such as bone fractures and skin injuries, that require unusual treatment were observed.

**Table 2:** Patient characteristic and number of WPAL trials.

**Level of injury (R/L)**	**Age**	**Sex**	**AIS**	**First year of trial**	**Number of WPAL trials**	**Frequency of WPAL trials per month**	**Current state**	**Reason for discontinuation**
**Cervical**								
C5/C5	44	Male	A	2018	3	3.0	End	Pain of upper limb
C5/C6	36	Male	B	2018	23	1.0	Ongoing	–
C6/C6	72	Male	B	2012	20	0.2	Ongoing	–
C6/C6	30	Female	B	2018	79	3.0	Ongoing	–
C6/T10	33	Male	A	2015	144	2.4	Ongoing	–
C7/C7	28	Male	A	2016	72	1.5	Ongoing	–
C7/C7	51	Male	B	2017	18	3.0	Ongoing	–
C7/C7	44	Male	C	2019	5	5.0	End	Social reasons
T1/C7	60	Male	B	2017	27	0.9	Ongoing	–
T1/C8	23	Male	B	2015	10	2.0	End	Hypertension
**Upper thoracic**								
T3/T3	36	Male	A	2019	13	3.3	Ongoing	–
T4/T4	63	Male	A	2013	9	0.4	End	Social reasons
T6/T6	60	Male	A	2007	39	1.6	End	Orthostatic hypotension
T6/T6	61	Female	A	2008	99	1.4	End	Social reasons
T6/T6	43	Male	A	2008	50	1.7	End	Cellulitis
**Lower thoracic**								
T7/T7	32	Male	A	2016	118	2.0	Ongoing	–
T7/T7	84	Male	C	2018	18	6.0	Ongoing	–
T8/T8	36	Male	A	2015	50	0.4	Ongoing	–
T8/T8	53	Male	A	2011	15	5.0	End	Social reasons
T9/T9	49	Male	A	2008	43	1.4	End	Pressure sore
T10/T10	20	Female	B	2012	4	0.5	End	Social reasons
T10/T10	22	Male	A	2014	14	1.6	End	Social reasons
T10/T10	64	Male	A	2015	208	3.9	Ongoing	–
T11/T11	54	Male	A	2012	29	4.8	End	Social reasons
T11/T11	51	Male	C	2012	7	1.4	End	Social reasons
T12/T12	42	Male	A	2007	179	1.3	Ongoing	–
T12/T12	33	Male	A	2008	25	0.8	End	Social reasons
T12/T12	40	Male	A	2014	10	0.7	End	Social reasons
T12/T12	26	Male	A	2014	38	0.9	Ongoing	–
T12/T12	40	Male	A	2015	252	3.9	Ongoing	–
**Lumbar**								
L1/L1	35	Female	C	2019	48	2.5	Ongoing	–

*WPAL, Wearable Power-Assist Locomotor*.

The mean number of WPAL trials for each patient was 55 (range 2–252) from 2007 to 2020. For the latest WPAL version, the mean number of WPAL trials for each patient was 51 (range 1–181). The person with the highest number of WPAL trials had an injury at T12 (AIS, A) and had the longest continuous walking distance and time. The most frequent reasons for discontinuation of WPAL gait training were social reasons unrelated to WPAL trials (10 patients), such as job-hunting and being busy with work, and discontinuation due to medical problems (5 patients). Twenty patients reached an FAC score of 4. The mean number of WPAL trials to reach an FAC score of 4 was 9 (median 8, range 2–22) in all patients and 13 (median 13, range 8–17) in the cervical group, 12 (median 12, range 8–16) in the upper thoracic group, 8 (median 8, range 2–22) in the lower thoracic group, and 6 in the lumbar group. However, 2 patients were excluded from these analyses due to missing data. The mean number of days taken to achieve an FAC score of 4 under the WPAL usage condition was 143 days (median 126, 16–555). Patients who reached an FAC score of 4 continued the WPAL trials. The mean number of continued WPAL trials was 66 (median 39, range 6–247). The number of continued WPAL trials varied among the patients due to factors unrelated to the WPAL trials, such as work. The mean duration following achievement of an FAC score of 4 was 1,221 days (median 763, 65–4,058) in December 2020.

### Gait Performance

**Figure 3:**
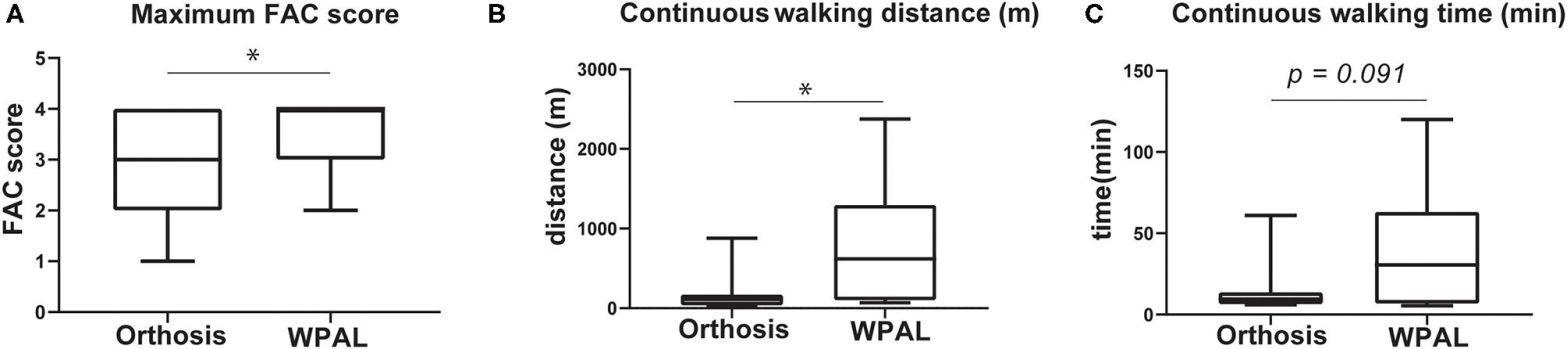
Comparisons of gait performance between the conventional orthosis and the WPAL. **(A)** Maximum FAC score, **(B)** continuous walking distance, and **(C)** continuous walking time. Each box represents 25–75% percentile and whiskers represent 5–95 percentile. Asterisk indicates statistically significant differences (*p* < 0.05). FAC, functional ambulation category; WPAL, Wearable Power-Assist Locomotor.

Table 3 and Figure 3 show the results of the gait performance in terms of the FAC scores and continuous walking distance and time when using the WPAL and the orthosis. Thirteen patients had improved FAC scores using the WPAL compared with those using the orthosis, whereas two patients had a decreased FAC score using the WPAL compared with those using the orthosis. In all patients using the WPAL, the continuous walking distance and time ranged from 5.0 to 2375.0 m (median 80 m) and from 0.5 to 120.0 min (median 10 min), respectively. Contrarily, when using the orthosis, the continuous walking distance and time ranged from 3.5 to 879.0 m (median 40 m) and from 1.9 to 61.0 min (median 6 min), respectively. For the 20 patients who achieved an FAC score of 4 using the WPAL, the continuous walking distance and time ranged from 20 to 2,375 m (median 99 m) and from 3 to 120 min (median 10 min), respectively. For using orthosis in the patients who achieved an FAC score of 4 using the WPAL, the continuous walking distance and time ranged from 9 to 879 m (median 40 m) and from 2 to 61 min (median 6 min), respectively. The FAC scores in the 26 patients in whom measurements were made for both the orthosis and WPAL, and the maximal continuous walking distance in eight patients who achieved an FAC score of 4 using both devices was significantly higher for the WPAL than for the orthosis (*p* = 0.005 and 0.012, respectively). However, the difference in the maximal continuous walking time in the eight patients who achieved an FAC score of 4 was not statistically significant between the WPAL and the orthosis (*p* = 0.091).

**Table 3:** Gait performance.

**Level of injury (R/L)**	**Maximum FAC score**		**Number of WPAL trials to reach a FAC score of 4**	**Consecutive walking distance (m)**		**Consecutive walking time (min)**	
	**Orthosis**	**WPAL**		**Orthosis**	**WPAL**	**Orthosis**	**WPAL**
**Cervical**							
C5/C5	1	2		–	–	–	–
C5/C6	4	3		–	20.3	–	11.1
C6/C6	1	2		–	–	–	–
C6/C6	3	3		3.5	50.4	2.7	18.2
C6/T10	3	4	Missing data	10.2	69.9	6.1	5.5
C7/C7	4	4	8	36.5	69.3	14.2	6.4
C7/C7	1	2		–	–	–	–
C7/C7	–	–		–	–	–	–
T1/C7	3	4	17	9.2	49.0	1.9	5.8
T1/C8	–	–		–	–	–	–
**Upper thoracic**							
T3/T3	–	4	13	–	154.1	–	14.5
T4/T4	2	3		–	5.0	–	0.5
T6/T6	2	4	16	20.0	30.0	5.0	4.5
T6/T6	2	4	10	40.0	80.0	5.0	9.5
T6/T6	4	4	8	40.0	80.0	8.0	8.0
**Lower thoracic**							
T7/T7	4	4	8	21.9	163.8	7.5	5.5
T7/T7	1	2		–	–	–	–
T8/T8	–	4	9	–	76.2	–	5.9
T8/T8	2	4	9	20.0	20.0	3.0	3.0
T9/T9	3	4	12	57.0	99.0	6.0	12.0
T10/T10	2	2		–	–	–	–
T10/T10	4	4	2	110.0	185.0	6.0	10.0
T10/T10	4	4	5	131.0	1052.0	12.0	51.0
T11/T11	3	4	7	40.0	76.0	5.0	9.5
T11/T11	4	4	Missing data	–	–	–	–
T12/T12	4	4	22	107.0	1095.0	6.0	64.0
T12/T12	3	4	8	44.0	220.0	6.0	18.0
T12/T12	4	3		–	–	–	–
T12/T12	4	4	5	186.0	1362.3	11.0	60.5
T12/T12	4	4	5	879.0	2375.0	61.0	120.0
**Lumbar**							
L1/L1	–	4	6	–	123.9	–	7.3

## Discussion

In this retrospective study that includes updated results from previous reports, all data were extracted from development research records from 2007 to 2020. The present findings confirm that the WPAL improves walking ability in patients with a wide range of SCIs compared with the orthosis, such as cervical SCI.

For the first time, we summarized the development history of the WPAL in this study. During this study period, the WPAL was updated seven times. The main updates were related to the safety and appearance of the device for the purpose of improved usability. Contrarily, the medial-type hip joint system without the trunk orthosis has not changed. Additionally, the location and number of motors have not changed since 2006. We believe that the development history would be helpful for devising a new gait-assisted robot (e.g., development order and required period of development). To the best of our knowledge, there is one report about the differences in the updates of the robots. Guanziroli et al. compared two different gait patterns using the Rewalk™ (ReWalk Robotics Ltd., Yokneam, Israel) between first- and second-generation software control; the latter had better synchronization between the hip and knee kinematics based on healthy kinematics and kinetics profiles for improvement of the quality of the gait pattern (Guanziroli et al., 2019). They reported an extension of the 6-min walking test and an improvement in the 10-m walking test.

The mean number of WPAL trials to reach an FAC score of 4 using a rolling walker was 9 in all patients and 13 in the cervical group, 12 in the upper thoracic group, 8 in the lower thoracic group, and 6 in the lumbar group. Some studies have reported the number of WRE trials required to walk with a walking aid and physical assistance (Esquenazi et al., 2012; Hartigan et al., 2015; Kozlowski et al., 2015; Guanziroli et al., 2019; Tsai et al., 2021) (Table 4). Esquenazi et al. reported that all the 12 patients with a motor-complete SCI (T3–T12 injury level) were able to walk independently without physical assistance using the ReWalk™ system (ReWalk Robotics Ltd., Yokneam, Israel) for at least 50–100 m continuously, for a period of at least 5–10 min continuously, after up to 24 trials (Esquenazi et al., 2012). Guanziroli et al. reported that 13 patients with a motor-complete SCI (T4–L4 injury level) required a mean of 22 trials to achieve independent walking using ReWalk™ with crutches (Guanziroli et al., 2019). Tsai et al. reported that eight patients with motor-complete SCIs (T1–T11 injury level) required a median of 30 (range 7–90) trials to achieve independent walking using ReWalk™ with crutches within a median of 111 days (range 87–210 days) (Tsai et al., 2021). Kozlowski et al. reported that 6 of 7 patients with motor-complete (4) or incomplete (3) SCIs (C4–T10 injury level) achieved walking using the Exso system (Exso Bionics, Richmond, CA, USA) with a front-wheeled walker or Lofstrand crutches and minimal assistance in a median of 8 trials, and 5 of them achieved walking with contact guard and close supervision assistance in a median of 15 trials (Kozlowski et al., 2015). Hartigan et al. reported three patients with motor-complete tetraplegia (C5–C7 injury level) who were able to walk after 5 WRE trials using a bilateral platform rolling walker with the minimal or moderate assistance of one therapist (Hartigan et al., 2015). In addition, six of the eight patients with motor-complete SCIs (T9–L1 injury level) were able to walk with supervised assistance using forearm crutches or a rolling walker (Hartigan et al., 2015). Regarding the level of injury, the WPAL was able to achieve walking independence with a rolling walker within a relatively small number of trials.

**Table 4:** Summary results of previous studies and the present study.

**References**	**Patients**	**Level of injury**	**Device**	**The degree of gait independence with the device**	**Number of gait training using the devices**
Esquenazi et al. (2012)	12 (motor-complete)	T3-12	ReWalk™	Independent	24 (max)
Guanziroli et al. (2019)	13 (motor-complete)	T4-L4	ReWalk™	Independent	22 (mean)
Tsai et al. (2021)	8 (motor-complete)	T1-11	ReWalk™	Independent	30 (median)
Kozlowski et al. (2015)	7 (4 motor-complete) (3 motor-incomplete)	C4-T10	Exso	Minimal assistance (6 patients) Contact guard and close supervision (5 patients)	8 (median) 15 (median)
Hartigan et al. (2015)	3 (motor-complete) 5 (4 motor-complete) (1 motor-incomplete) 8 (7 motor-complete) (1 motor-incomplete)	C5-7 T1-8 T9-L1	Indego	Minimal/ moderate (3 patients) Supervision (2 patients) Minimal assistance (3 patients) Supervision (6 patients) Minimal assistance (2 patients)	5
Present study	31 (motor-complete)	C5-L1	WPAL	Independent (20 patients)	9 (mean)^*^

* *The mean number of WPAL trials to reach an FAC score of 4 using a rolling walker. WPAL, Wearable Power-Assist Locomotor; FAC, functional ambulation category*.

The maximum continuous walking time and distance using the WPAL were 120 min and 2,375 m, respectively. The maximum run time of WPAL is 120 min owing to the battery capacity. This result suggests that the WPAL has sufficient walking performance for it to be utilized at home and in the community. van Dijsseldonk et al. (2020) investigated the number of WREs used in home and community environments in 14 patients with SCIs at an injury level of T4 to L1 (van Dijsseldonk et al., 2020). This previous study reported that the estimated median active time is 46 (range 19–84) min, during which the median estimated total distance covered is 243 (range 22–1,367) m, and the median estimated maximal distance covered without rest is 120 (range 12–1,125) m (van Dijsseldonk et al., 2020).

In the present study, 10 patients with cervical SCIs practiced the WPAL gait. All patients with cervical SCIs who reached an FAC score of 4 in the WPAL gait had longer continuous walking distances; however, two patients had shorter continuous walking time compared with those using an orthosis. The results of the longer walking distance with shorter walking time for WPAL than for orthosis suggests that WPAL walking requires a more quick and constant lateral weight shift than orthotic walking. A previous study reported that a patient is required to make a quick and constantly lateral weight shift with WPAL motion during the WPAL gait. This rhythmic lateral weight shift is produced by the lateral force using the upper limbs (Tanabe et al., 2017). Even with other WREs, independent rhythmic lateral weight shifts during walking are difficult for patients with cervical SCIs. Many previous studies reported that other WREs require assistance for walking in patients with cervical SCIs (Hartigan et al., 2015; Kozlowski et al., 2015; Benson et al., 2016; Birch et al., 2017). A previous study reported that two patients with cervical SCIs (a motor-complete C8 and a motor-incomplete C4) were able to walk over 100 m using an Ekso powered exoskeleton with supervision or minimal assistance (Kozlowski et al., 2015). Benson et al. (2016) reported that one patient with motor-incomplete C7 lesions, who could walk without using an exoskeleton, was able to walk up to 91 m in a 6-min walk test using the ReWalk™ (Benson et al., 2016). Hartigan et al. (2015) reported that patients with three motor-complete cervical SCIs (one C5 and two C6 lesions) were able to walk an average distance of 64 m in a 6-min walk test using an Indego exoskeleton (Parker Hannifin Corporation) and a bilateral platform rolling walker with minimal or moderate assistance from one therapist (Hartigan et al., 2015). Birch et al. (2017) reported that five patients with cervical SCIs with motor-incomplete injury (three C4 and two C6 lesions) were able to perform a timed up and go test in a mean of 302 s (95% CI ± 49.6 s) with one assistant for three patients and two assistants for two patients using the REX robotic exoskeleton (Rex Bionics) (Birch et al., 2017). In the present study, patients with C5–C6 lesions did not reach an FAC score of 4 using the WPAL, on equality with using other WREs. However, patients with C7 lesions, even with motor-complete SCIs, reached an FAC score of 4 even in patients with motor-complete because the WPAL has a high standing stability, which is a structural characteristic similar to medial-type orthoses (Saitoh et al., 1996; Tanabe et al., 2013b; Koyama et al., 2016).

For using WPAL, the number of gait training to achieve independent gait was tended to less than other robots. In addition, WPAL tended to achieve independent gait in patients with cervical SCI compared with other robots. However, previous studies determined the number of gait training in advance during study periods and reported the degree of gait independence after completing total sessions of gait training. In future studies, it is necessary to compare the number of gait training to acquire independent walking with the gait-assisted robot among different robots in the same patients with SCI, rather than studies with a predetermined number of gait training.

In this study, the number and frequency of WPAL trials differed among patients. A previous study suggested that the time required to learn to safely walk with WREs is affected by the learning capacity, level of injury and completeness of SCI, and the user's strength and endurance levels (Kandilakis and Sasso-Lance, 2021). In the future, we need to clarify the rate of achievement of independent walking by configuring the previous number and frequency of WPAL trials. Moreover, the accumulation of clinical trials using WREs helps to elucidate the optimal number of training sessions and the frequency per week.

In this study, patients were able to continue the WPAL trail even after reaching an FAC score of 4 if they wished. In the future, the long-term effects of habitual walking using the WPAL on physical functions should be examined. Prolonged sitting in a wheelchair is associated with an increased risk of all-cause mortality (Rezende et al., 2016). Patients with SCIs commonly engage in less physical activity compared with healthy adults (Haisma et al., 2006; van den Berg-Emons et al., 2010). According to the physical activity guidelines, patients with SCIs should engage in at least 30 min of moderate- to vigorous-intensity aerobic exercise three times per week for cardiometabolic health benefits (Martin Ginis et al., 2018). Some studies reported that walking with WREs has numerous beneficial effects on pulmonary function (Xiang et al., 2021), bladder function (Chun et al., 2020), and sitting balance (Tsai et al., 2021). Habitual walking exercises using the WPAL three times a week for a long time may provide health benefits for patients with SCIs.

Long-term use in the community and its effects should be examined. Another robot is already being used in daily life situations (van Dijsseldonk et al., 2020). Further refinement of the WPAL will enable its long-term use in homes. The common prevent factor for long-term use is that a wheelchair is a very safe and comfortable mobility device for daily transportation in patients with SCI. Because the homes of wheelchair users are remodeled to make them accessible for wheelchair users, they can live without walking or standing. However, the robots have the advantage when patients with SCI perform cooking, washing, and cleaning (windows and shelves that cannot be reached on the wheelchair). Kandilakis et al. reported that the goal of robotic exoskeleton use is not to replace a wheelchair, but to create a supplemental means of mobility, exercise, or activities of daily living (Kandilakis and Sasso-Lance, 2021). We also believe that the alternate usage between robots and wheelchairs is the goal in the near future. A beneficial point of the WPAL is that it is possible to move using a wheelchair with the WPAL attached to their lower limbs because the WPAL has motors installed between both the lower limbs and no trunk support orthosis.

Further important development points for all robots, such as the WPAL, are the stair climbing, the lateral movement in a standing position, the ease of transporting the robots and the walking aids, the lightweight of the robots, the ease of donning/doffing them, the easy maintenance, high sound/waterproofing property, and fall prevention or detection system. For these, it is necessary to improve elemental technologies, such as the motors and the materials used for the robots. Lastly, we should examine whether the WPAL can improve or recover motor function in patients with motor-incomplete injury by partial motor assistance rather than complete motor assistance.

## Data Availability Statement

The original contributions presented in the study are included in the article/supplementary material, further inquiries can be directed to the corresponding author.

## Ethics Statement

The studies involving human participants were reviewed and approved by Institutional Review Board of Fujita Health University. The patients/participants provided their written informed consent to participate in this study.

## Author Contributions

SK performed the data collection, analysis, writing original draft and review, editing, designing the research, and project administration. ST designed the research, writing—original draft, writing—review, and editing. TG and YT participated in data collection, analysis, and writing original draft. MK, ES, YO, and SH participated in data collection, writing original draft, writing review and editing, directed the research, project administration, and supervision. All authors contributed to the article and approved the submitted version.

## Funding

This work was supported by a grant-in-aid of Basic Technology Development for Practical Application of Human Support Robots [8068149], Practical Development of Industrial Technology [8080694] by New Energy and Industrial Technology Development Organization (NEDO), and the JSPS KAKENI, Grant Number 20K11223.

## Conflict of Interest

The authors declare that the research was conducted in the absence of any commercial or financial relationships that could be construed as a potential conflict of interest.

## Publisher's Note

All claims expressed in this article are solely those of the authors and do not necessarily represent those of their affiliated organizations, or those of the publisher, the editors and the reviewers. Any product that may be evaluated in this article, or claim that may be made by its manufacturer, is not guaranteed or endorsed by the publisher.

## Abbreviations

AIS: American Spinal Injury Association Impairment Scale
FAC: functional ambulation category
SCI: spinal cord injury
WPAL: Wearable Power-Assist Locomotor
WRE: wearable robotic exoskeletons.
